# From Cloning Vectors to Phage Display: Engineering Principles and Translational Applications of Bacteriophage Display Platforms

**DOI:** 10.3390/ijms27167124

**Published:** 2026-08-08

**Authors:** Tingting Hong, Nan Chen, Yingli Yang, Yao Wang, Yao Yao, Caihong Zheng

**Affiliations:** Department of Pharmacy, Women’s Hospital, School of Medicine, Zhejiang University, Hangzhou 310006, China; hongtingting2026@163.com (T.H.); chennan2719@zju.edu.cn (N.C.); yangyingli0110@163.com (Y.Y.); jidantou@outlook.com (Y.W.)

**Keywords:** bacteriophages, phage display, antibody discovery, vaccine design, phage engineering, biopanning

## Abstract

Bacteriophage technologies have evolved from classical cloning vectors into programmable platforms for genome engineering and molecular selection. Phage display couples a surface-presented binding phenotype to its encoding genotype, enabling iterative selection, sequence recovery, and optimization of peptides, antibody fragments, and other protein binders. This review links phage morphology, genome organization, infection strategy, host defense, and engineering method to practical platform choice. It compares λ, N15, M13, and T7 systems; examines biopanning bias and candidate developability; and evaluates artificial intelligence-assisted, sequencing-guided, and structure-guided workflows. Vaccine applications are considered alongside constraints arising from anti-phage immunity, antigen density, route of administration, and repeat dosing. Progress will depend on experimentally validated closed-loop workflows that integrate library design, selection, high-throughput analytics, structural characterization, and early manufacturability assessment.

## 1. Introduction

**Figure 1:**
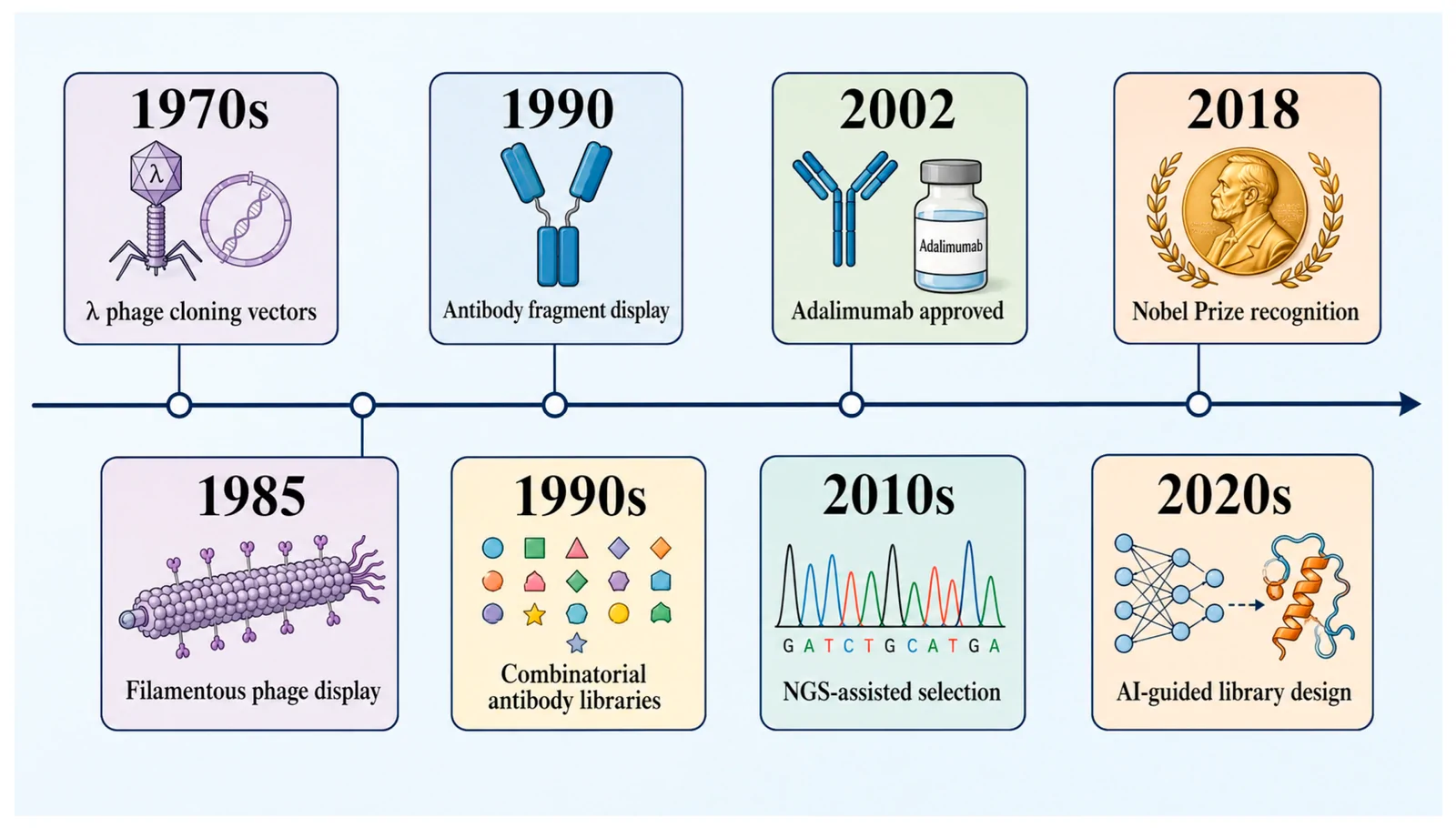
Historical evolution of bacteriophage vectors and phage-display technologies. The timeline traces the transition from λ phage cloning vectors in the 1970s [4] to filamentous phage display in 1985 [1] and antibody-fragment display in 1990 [2]. Subsequent milestones include the expansion of combinatorial antibody libraries in the 1990s, approval of adalimumab in 2002 [5], integration of next-generation sequencing (NGS) into selection workflows in the 2010s [6,7], Nobel Prize recognition of phage display in 2018 [3], and the emergence of artificial intelligence (AI)-guided library design in the 2020s [8]. Abbreviations: AI, artificial intelligence; NGS, next-generation sequencing. This figure was originally created by the authors using Adobe Illustrator (version 30.6).

Bacteriophages occupy a distinctive position in biotechnology because intact particles can function as genetic vehicles or physically selectable display scaffolds. Classical λ- and N15-derived systems enabled genomic and complementary DNA (cDNA) library construction and stable maintenance of difficult inserts, whereas filamentous phages made surface display directly accessible. In 1985, George P. Smith showed that foreign peptides could be displayed on filamentous phage, establishing genotype–phenotype linkage in this format [1]. McCafferty and colleagues subsequently extended the principle to antibody variable domains [2]. The 2018 Nobel Prize in Chemistry recognized the impact of phage display on directed evolution [3]. Figure 1 places these advances in the broader progression from λ cloning vectors to sequencing- and artificial intelligence (AI)-assisted phage-display workflows.

Unlike direct antibacterial phage therapy, most phage-display campaigns use phage as a discovery vehicle and deliver a defined peptide, antibody, or protein as the eventual product. This separation generally simplifies sequence definition and downstream standardization, although the selection process itself remains sensitive to library construction, propagation, and assay design [9,10]. Library size alone is therefore a poor proxy for discovery quality. Display valency, coat-protein context, target presentation, washing and elution conditions, clone growth, sequencing depth, and early developability filters jointly determine whether an enriched clone can become a useful reagent or therapeutic lead [6,9,10].

**Figure 2:**
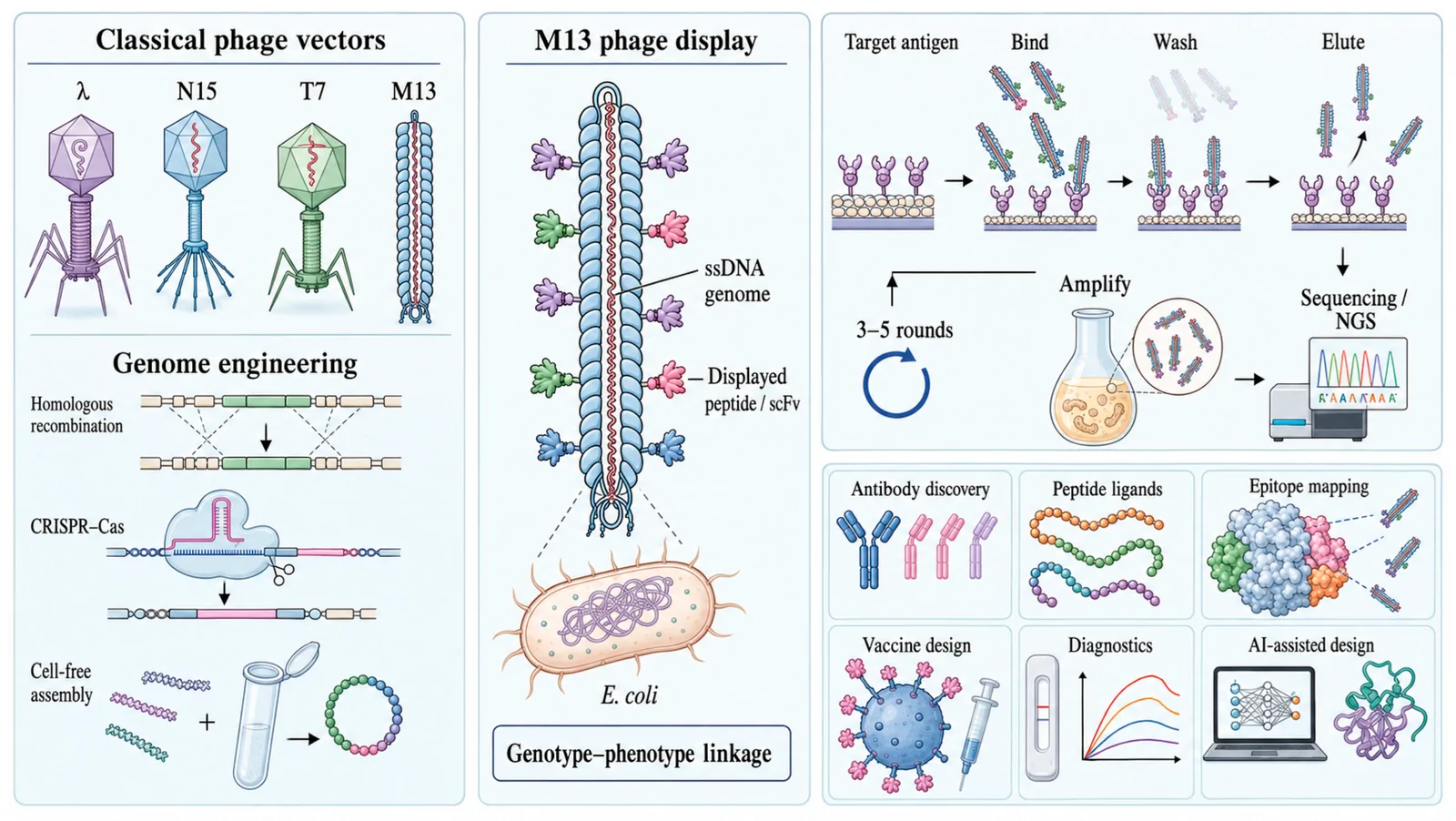
Integration of classical phage vectors, M13 phage display, biopanning, and translational applications. Lambda (λ), N15, T7, and M13 phages provide complementary platforms for DNA cloning, maintenance of large DNA fragments, protein expression, and surface display. Homologous recombination, CRISPR–Cas editing, and cell-free assembly enable construction and reprogramming of phage genomes. In the M13 platform, a displayed peptide or single-chain variable fragment (scFv) is physically linked to the packaged single-stranded DNA (ssDNA) that encodes it, thereby establishing genotype–phenotype linkage. During biopanning, a diverse library is exposed to an immobilized target; non-binders are removed by washing, whereas retained phages are eluted, amplified in *Escherichia coli*, and subjected to three to five enrichment rounds before sequencing or next-generation sequencing (NGS). Enriched binders can support antibody discovery, peptide-ligand identification, epitope mapping, vaccine design, diagnostics, and AI-assisted molecular design. Abbreviations: AI, artificial intelligence; CRISPR–Cas, clustered regularly interspaced short palindromic repeats–CRISPR-associated system; NGS, next-generation sequencing; scFv, single-chain variable fragment; ssDNA, single-stranded DNA. This figure was originally created by the authors using Adobe Illustrator (version 30.6).

Recent reviews have examined peptide-display technologies, protein-drug discovery, and phage vaccine platforms separately [9,10,11]. Here, these topics are integrated from a platform-selection perspective: phage morphology, replication, and bacterial defense are related to vector choice, and classical and alternative display systems are evaluated alongside selection bias, antibody developability, AI-assisted design, and vaccine-specific constraints. The discussion proceeds from phage biology and vector engineering to M13-based display, biopanning, antibody discovery, vaccine design, and immunological applications. Figure 2 provides an integrated overview of classical phage vectors, genome-engineering strategies, genotype–phenotype linkage in M13 display, iterative selection, sequence recovery, and major translational outputs.

## 2. Biology of Bacteriophages

**Figure 3:**
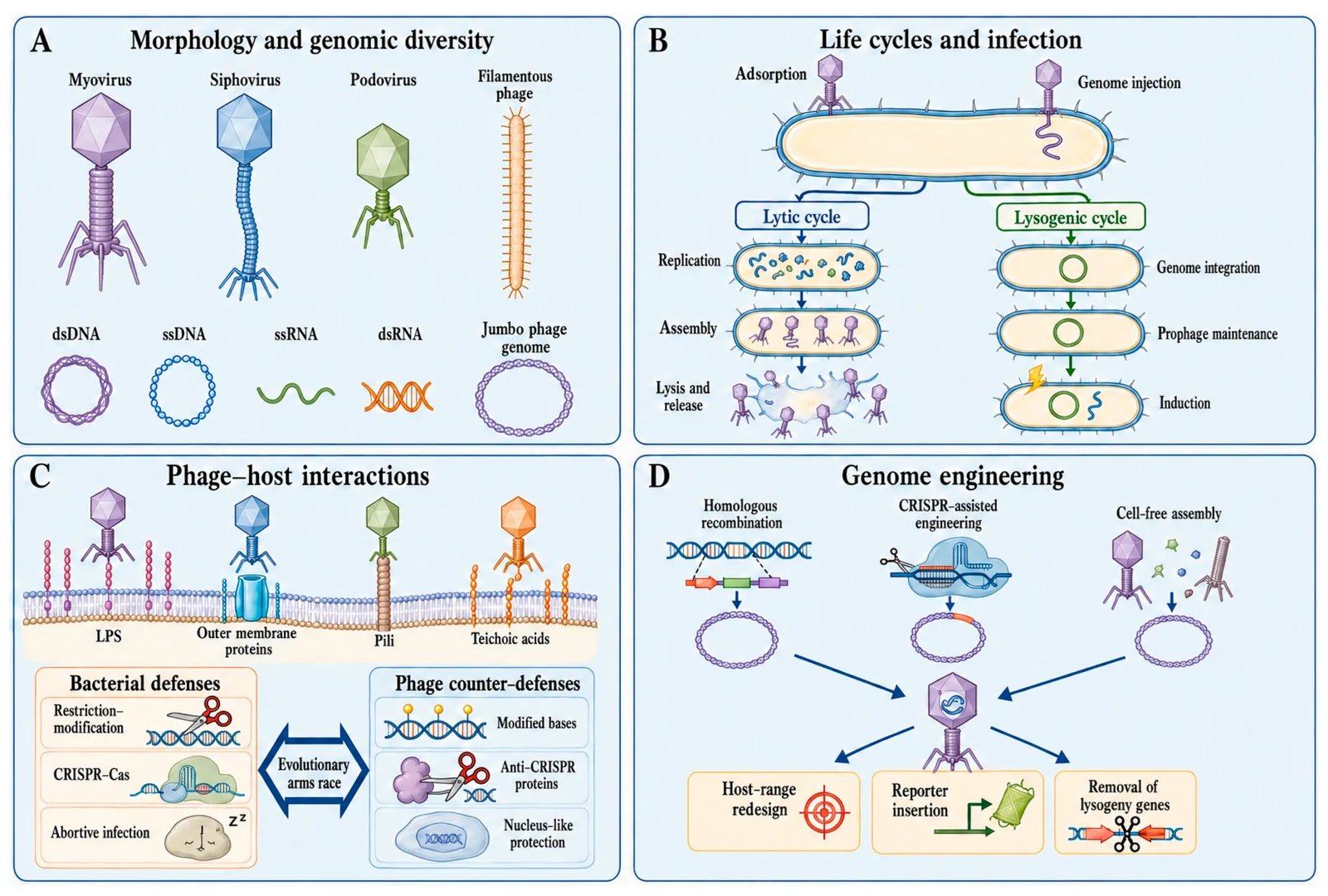
Morphological and genomic diversity, infection cycles, host interactions, and engineering of bacteriophages. (**A**) Representative myovirus-like, siphovirus-like, and podovirus-like tailed morphotypes and a filamentous phage are shown alongside double-stranded DNA (dsDNA), single-stranded DNA (ssDNA), single-stranded RNA (ssRNA), double-stranded RNA (dsRNA), and jumbo-phage genomes. (**B**) Following adsorption and genome injection, phages may enter a lytic cycle involving replication, assembly, and host-cell lysis or a lysogenic cycle involving genome integration, prophage maintenance, and stress-induced entry into lytic growth. (**C**) Phage adsorption is mediated by bacterial surface structures, including lipopolysaccharide (LPS), outer-membrane proteins, pili, and teichoic acids. Bacterial defenses such as restriction–modification, CRISPR–Cas, and abortive infection interact with a diverse phage counter-defense repertoire that includes modified bases, anti-CRISPR proteins, and nucleus-like protective compartments, collectively driving an evolutionary arms race. (**D**) Homologous recombination, CRISPR-assisted engineering, and cell-free assembly can be used to redesign host range, insert reporters, or remove lysogeny-associated genes. Abbreviations: CRISPR–Cas, clustered regularly interspaced short palindromic repeats–CRISPR-associated system; dsDNA, double-stranded DNA; dsRNA, double-stranded RNA; LPS, lipopolysaccharide; ssDNA, single-stranded DNA; ssRNA, single-stranded RNA. This figure was originally created by the authors using Adobe Illustrator (version 30.6).

Bacteriophage biology defines the feasible engineering space. Particle architecture and genome type (Figure 3A), infection strategy (Figure 3B), receptor recognition and the evolutionary arms race between phages and their hosts (Figure 3C), and the available genome-engineering method (Figure 3D) influence cargo tolerance, host requirements, display valency, and recovery of viable particles. Bacterial-defense–phage-counter-defense systems further determine whether an engineered genome can be recovered and propagated. These variables guide the choice between cloning, non-lytic display, lytic expression, host-range engineering, and cell-free assembly.

### 2.1. Morphology and Genomic Diversity

Bacteriophages comprise tailed, non-tailed, and filamentous particles. Among tailed phages in the class Caudoviricetes, adsorption and genome delivery depend on distal structures such as tail fibers, tail spikes, baseplates, and terminal receptor-binding proteins rather than on a uniform “tail” function. The familiar myovirus-like, siphovirus-like, and podovirus-like terms are now informal morphotypes; the former families Myoviridae, Siphoviridae, and Podoviridae have been abolished, and formal taxonomy is based principally on genomic and core-protein relationships [12,13].

Phage genomes may consist of double-stranded DNA (dsDNA), single-stranded DNA (ssDNA), single-stranded RNA, or double-stranded RNA, and range from a few kilobases to several hundred kilobases. Members of the order Crassvirales, for example, typically carry 145–192-kb genomes [14]. Lytic genomes emphasize replication, assembly, and lysis modules, whereas temperate genomes also encode integration and repression functions. CTXΦ of *Vibrio cholerae* illustrates repressor-controlled lysogeny [15]. Acquisition of auxiliary metabolic genes and exchange of functional modules contribute to ecological adaptation and genomic mosaicism [16,17].

This diversity supplies engineering parts but also imposes compatibility rules. CRISPR–Cas editing can enrich intended changes in susceptible phage families, whereas directed evolution of receptor-binding proteins can alter host specificity [18,19,20,21]. Neither approach is portable without testing the phage genome chemistry, replication compartment, host genotype, and fitness cost of the edit.

Platform choice should follow the intended product. Filamentous ssDNA phages are useful when non-lytic secretion and coat-protein fusion are required; compact lytic dsDNA phages support rapid particle production or capsid display; and N15-derived linear-plasmid systems can stabilize inserts that are difficult to maintain in conventional circular plasmids [22]. Larger genomes may provide more cargo space but also increase the burden of assembly, rebooting, and sequence verification.

### 2.2. Life Cycles and Infection

Phage replication is commonly described as lytic or temperate. The distinction determines not only biological outcome but also acceptable engineering and biosafety strategies.

Strictly lytic phages replicate, assemble progeny, and lyse the host; this behavior supports direct bacterial killing and rapid particle production. For example, phage P509 lyses carbapenem-resistant *Klebsiella pneumoniae* in vitro [23]. Temperate phages can instead establish prophages or persistent replicons through regulated lysis–lysogeny switches, as illustrated by the CI/MOR system of *Lactococcus lactis* phage TP901-1 and the gp07 anti-repressor of *Staphylococcus aureus* phage Φ11 [24,25].

Environmental cues can shift these decisions: Mu-like phages of *Yersinia pestis* favor lysogeny at 26 °C and lytic growth at 37 °C [26]. For therapeutic or production uses, lysogeny genes, virulence determinants, and transduction potential require explicit screening because temperate phages can alter host phenotypes or mobilize genetic modules [27,28]. Strictly lytic starting points or validated deletion of lysogeny functions are therefore preferred when bacterial killing is the objective.

### 2.3. Phage Host Interactions

Phage–host contact begins with receptor recognition, but the relevant structures differ among phages. Tail fibers, tail spikes, baseplate proteins, and terminal tail complexes can recognize lipopolysaccharide (LPS), outer-membrane proteins, pili, teichoic acids, or related surface molecules [29,30,31]. Reversible attachment may be followed by conformational changes that commit the particle to genome delivery, as illustrated by the T5 Pb5–FhuA complex [31].

Host range can arise from multiple receptor-binding proteins or from a single protein with broad recognition. GSP004, for example, uses ORF208 to recognize O-antigens in *Salmonella* and *Escherichia coli* O157:H7 [32]. Directed evolution has expanded the host spectrum of resistant *Klebsiella pneumoniae* strains [33]. Some jumbo phages add a second layer of protection: phiKZ builds a nucleus-like compartment in *Pseudomonas aeruginosa* that shields replicating DNA [34].

Bacteria counter phage infection through several mechanistically distinct layers. Restriction–modification systems cleave inadequately modified foreign DNA, whereas CRISPR–Cas systems use spacer-derived RNAs to recognize previously encountered nucleic acids [35]. Abortive-infection and related defense systems can arrest growth or kill an infected cell. Two examples are the phage anti-restriction-induced system (PARIS)-associated nuclease AriB, which cleaves transfer RNA (tRNA) and suppresses translation, and cyclic GMP–AMP (cGAMP) signaling, which activates antiviral defenses [36,37].

Phages counter these defenses through mechanisms that include anti-CRISPR proteins, modified bases, and DNA-protective compartments [19,34,38]. These interactions drive phage–host coevolution and can also be exploited as counterselection during genome editing.

Bacterial defense affects both editing yield and display-library composition. Restriction-active or CRISPR-active hosts may remove newly assembled genomes before infectious particles are recovered, and abortive-infection systems can reduce burst size or select escape variants. In M13 libraries, host genotype, infectivity, secretion burden, and helper-phage compatibility further shape clone recovery during amplification [7,9]. The production strain, defense genotype, and rescue protocol should therefore be documented, and library diversity should be remeasured after propagation.

### 2.4. Genome Engineering

Phage genome engineering converts biological knowledge into defined changes: receptor-binding proteins can be altered to retarget hosts; reporters or antibacterial payloads can be inserted; and integrases, repressors, or other lysogeny-associated genes can be removed. Each design must preserve essential genome organization and packaging constraints.

Homologous recombination, CRISPR-assisted counterselection, cell-free genome assembly, and genome rebooting can introduce defined changes [18,19,20,39,40]. Sequence confirmation should be followed by infectivity, host-range, stability, and safety testing, because a correctly edited genome does not by itself establish a useful phenotype.

CRISPR–Cas systems can deplete wild-type backgrounds and enrich recombinant phages [18,19,20]. Golden Gate assembly, cell-free transcription–translation (TXTL) rebooting, and all-cell-free construction can reduce dependence on intracellular editing and accelerate library generation [39,40].

No genome-engineering method is uniformly efficient across phage families. Recombination frequency and CRISPR susceptibility depend on both phage and host; modified bases or nucleus-like compartments can restrict nuclease access; and secondary recombination, off-target cleavage, or compensatory mutations can obscure genotype–phenotype assignment. Large edits may reduce fitness or be lost during serial passage. Scale-up also requires controlled host banks, complete genome sequencing, impurity control, containment, and traceable documentation. These constraints should inform platform selection at the outset.

#### 2.4.1. Homologous Recombination

Lambda Red recombineering introduces a homologous donor into the production host to delete, insert, or replace a defined phage sequence. The method has been applied to the lytic phage T7 [41]. The splitting, modifying, assembling, and rebooting (SMART) workflow extends this principle by splitting the T7 genome into overlapping fragments, cloning and modifying the fragments in *Escherichia coli*, and then reassembling and rebooting the complete genome. In the reported implementation, the approach produced genome-reduced T7 chassis variants and freed approximately 10% of the genome for additional functional modules [42].

#### 2.4.2. CRISPR-Assisted Engineering

CRISPR-assisted editing cleaves the wild-type background and thereby enriches homologous recombinants that escape targeting. CRISPR–Cas9 editing of *Staphylococcus aureus* phage K, for example, enabled efficient construction of defined mutants, including reporter phages for bacterial detection [20].

DNA-targeting CRISPR systems may fail when phage DNA is chemically modified or sequestered in a proteinaceous compartment. RNA-targeting Cas13a avoids this barrier by cleaving phage transcripts while homologous recombination supplies the desired edit. Cas13a has been used to edit hydroxymethylcytosine-containing T4 [19] and the nucleus-forming jumbo phage ΦKZ [43]. Processive Cas3 has likewise supported targeted deletion, insertion, and replacement in virulent *Pseudomonas* phages [44].

#### 2.4.3. Cell-Free Assembly and High-Throughput Screening

Two complementary host-bypass strategies have emerged for phages that are toxic or difficult to edit intracellularly. Yeast-based platforms assemble and modify phage genomes by homologous recombination before transfer to a bacterial host for rebooting [45]. Cell-free approaches instead assemble genomes in vitro and recover particles by cell-free transcription–translation (TXTL) [39].

Golden Gate assembly is well suited to ordered reconstruction of large phage genomes. Polymerase chain reaction (PCR)-amplified or synthetic fragments are flanked by Type IIS restriction sites and user-defined overhangs. Because cleavage occurs outside the recognition sequence, a one-pot restriction–ligation reaction can join the fragments in a prespecified order without retaining the restriction sites [46]. Reliable assembly requires removal or domestication of internal Type IIS sites, selection of overhangs with low cross-reactivity, and placement of junctions away from essential coding, regulatory, and packaging elements. Data-optimized Golden Gate design has assembled a 40-kb T7 genome from 52 fragments, illustrating the scale attainable when junctions are selected systematically [47]. An assembled genome can then be rebooted in a TXTL reaction. The bacterial lysate supplies transcription, translation, virion assembly, and genome-packaging functions; a correctly assembled genome containing the required cis-acting elements can therefore yield infectious particles that are quantified by plaque assay [39]. Kristensen et al. combined Golden Gate assembly with TXTL rebooting of T7 and incorporated short DNA barcodes to track genome variants. Sequencing barcode abundance before and after rebooting or selection revealed changes in library diversity, variant loss, and condition-specific enrichment [39]. PHage Engineering by In vitro Gene Expression and Selection (PHEIGES) removed the remaining in-cell assembly step: PCR-amplified fragments were assembled and expressed directly in an *Escherichia coli* TXTL system. The workflow generated recombinant T7 particles and supported screening of a tail-fiber mutant library for altered host specificity [40]. Assembly and rebooting establish that an engineered genome can produce infectious particles; they do not establish therapeutic performance. Candidates still require complete sequence verification, host-range and stability testing, safety assessment, and validation in the intended biological context. Computational design can reduce experimental search space. PhagePromoter, for example, predicts promoter activity and can help select insertion sites and expression timing for engineered payloads [48]. Predictions should nevertheless be confirmed in the relevant phage–host system.

Together, these methods enable host-range modification, payload delivery, reporter expression, and removal of safety-related genes. Before translation, each construct requires complete sequence verification, genetic-stability testing over production-relevant passages, and phenotypic confirmation in the intended host. For scale-up, the engineered phage and production host must be treated as a coupled process. Critical attributes include yield, lysis or secretion kinetics, residual host DNA and endotoxin, genetic homogeneity, and lot-to-lot potency. Regulatory requirements also depend on product use: a phage employed only as a discovery reagent requires a narrower control strategy than a particle administered to patients, for which identity, purity, biodistribution, immunogenicity, and shedding become relevant.

## 3. Phage Vectors and Display Platforms

**Figure 4:**
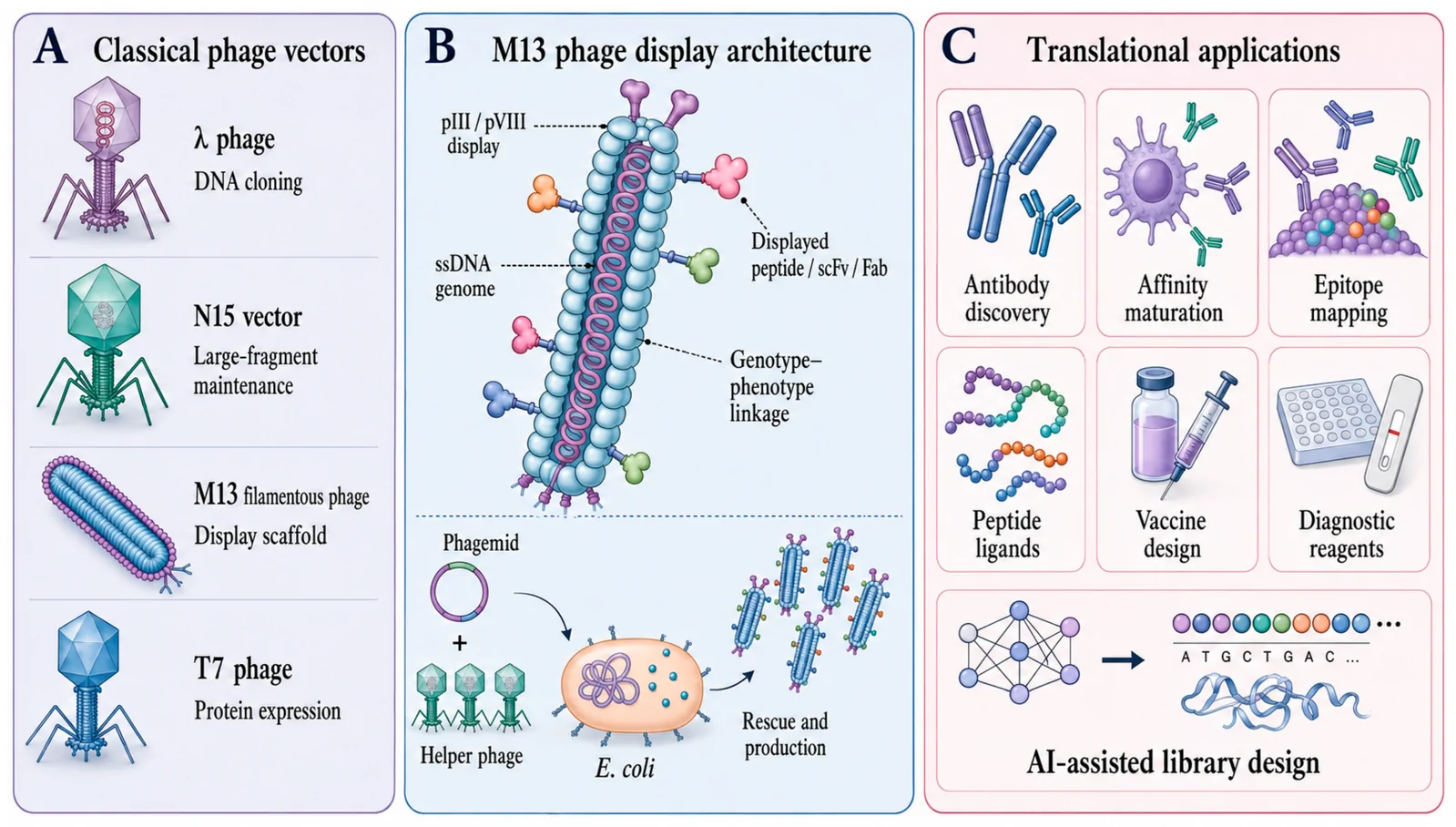
Classical phage vector platforms, M13 phage-display architecture, and major translational outputs. (**A**) Lambda (λ), N15, M13, and T7 phages have been adapted for DNA cloning, large-fragment maintenance, surface display, and protein expression, respectively. (**B**) In M13 display, peptides, single-chain variable fragments (scFvs), or antigen-binding fragments (Fabs) are fused to coat proteins such as pIII or pVIII, while the corresponding coding sequence is retained within the packaged ssDNA genome. This physical coupling underlies genotype–phenotype linkage. Phagemids are introduced into *Escherichia coli* and rescued with helper phage to produce display particles. (**C**) Major applications include antibody discovery and affinity maturation, epitope mapping, peptide-ligand screening, vaccine design, diagnostic-reagent development, and AI-assisted library design. Abbreviations: AI, artificial intelligence; Fab, antigen-binding fragment; scFv, single-chain variable fragment; ssDNA, single-stranded DNA. This figure was originally created by the authors using Adobe Illustrator (version 30.6).

Classical phage vectors solve different experimental problems. λ and N15 support cloning or stable maintenance of DNA; T7 couples a lytic genome with strong transcription and capsid-display options; and M13 provides non-lytic filamentous propagation and coat-protein display. These differences are summarized in Table 1 and Figure 4A.

### 3.1. Classical Phage Vectors

λ is a linear dsDNA phage with a well-defined packaging window and historical utility for genomic and cDNA libraries; replacement vectors typically accommodate approximately 10–25 kb of foreign DNA [4]. The N15-derived pJAZZ system uses TelN/tos functions to maintain hairpin-ended linear replicons and is useful for repetitive, unstable, or otherwise difficult DNA fragments [22].

M13 carries a circular ssDNA genome that forms a double-stranded replicative intermediate and exits *Escherichia coli* without lysis. Foreign peptides or antibody fragments can be fused principally to the minor coat protein pIII or the major coat protein pVIII, making M13 and phagemid derivatives the dominant affinity-selection platforms [9].

T7 is a lytic dsDNA phage whose RNA polymerase/promoter system supports strong transcription. T7 capsid-fusion formats can display peptides and proteins, including sequences that are poorly secreted through the *Escherichia coli* periplasm. Cargo and display tolerance depend on the vector locus and valency, so T7 should be treated as a family of platform-specific designs rather than as a fixed-capacity system [9]. Approximate capacities are vector- and construct-dependent and should not be interpreted as absolute packaging limits [4,9,22].

### 3.2. Phage-Display Principles

In M13 display, a peptide or antibody-fragment gene is fused to a coat-protein gene, most often pIII or pVIII. Each particle carries the coding sequence and presents the corresponding product, allowing a binding phenotype to be recovered as DNA [9]. Natural bacterial host recognition remains mediated by the phage infection machinery; the engineered fusion mediates binding to the experimental target. The M13 display architecture and its physical genotype–phenotype linkage are illustrated in Figure 4B.

A library is exposed to purified antigen, cells, tissue, or another target format. Unbound particles are removed, retained particles are eluted and amplified, and the process is repeated under adjusted stringency. Selection can incorporate negative depletion, competitive ligands, or off-rate challenges. Sequencing then identifies enriched clones for independent expression and testing [5,9].

**Figure 5:**
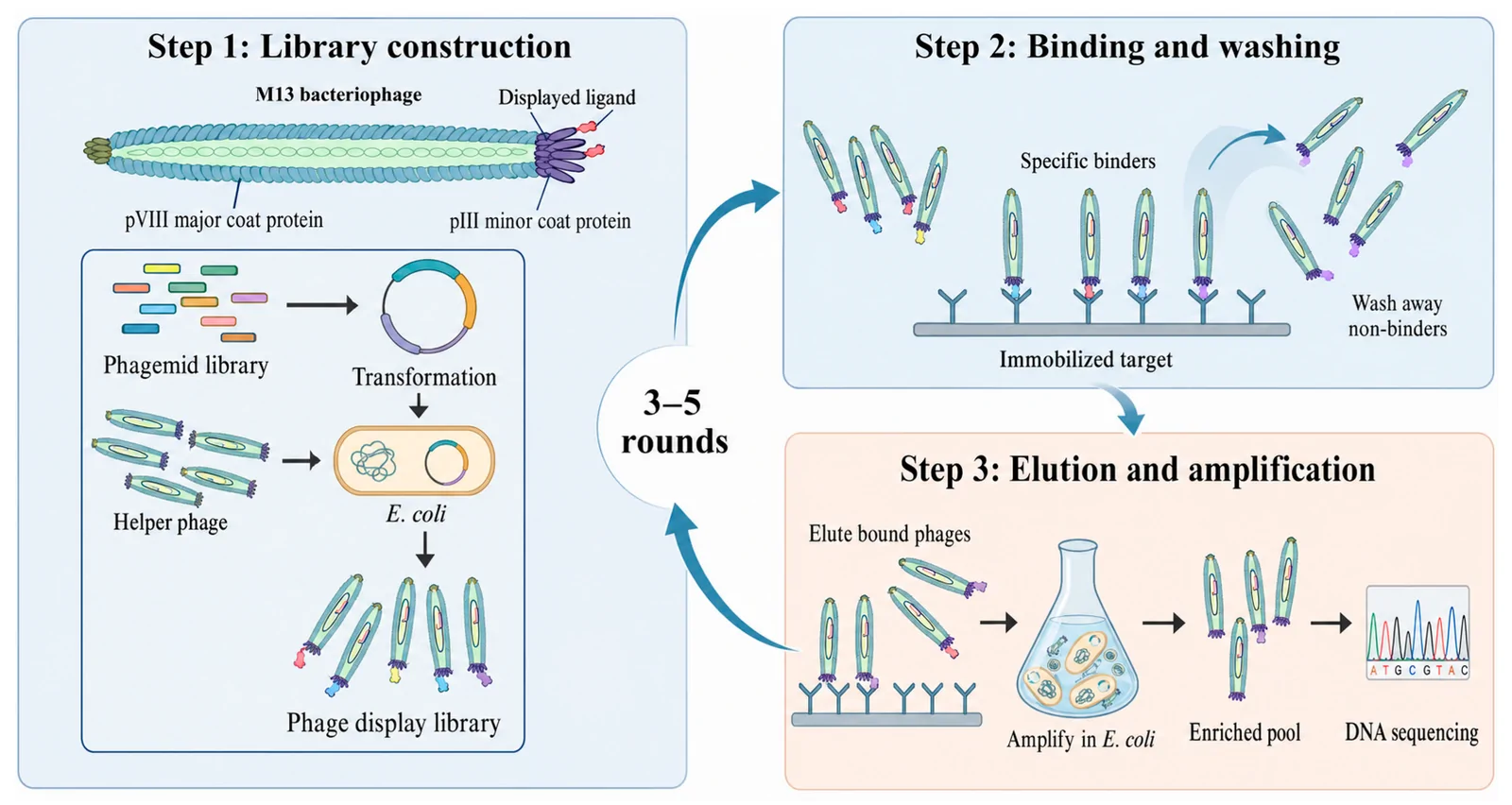
M13 phage-display library construction and iterative biopanning. Step 1: DNA sequences encoding peptides, single-chain variable fragments (scFvs), or antigen-binding fragments (Fabs) are cloned into phagemids as fusions to a coat-protein gene, commonly pIII. Following transformation of *Escherichia coli* and helper-phage rescue, the resulting particles display the encoded ligand while carrying its cognate DNA sequence. Step 2: The library is incubated with an immobilized target, and non-binding particles are removed by washing. Step 3: Target-bound phages are eluted and amplified in *Escherichia coli* to generate an enriched pool. Repetition for three to five rounds progressively enriches specific binders, which are then identified by DNA sequencing. Abbreviations: Fab, antigen-binding fragment; pIII, minor coat protein III; scFv, single-chain variable fragment. This figure was originally created by the authors using Adobe Illustrator (version 30.6).

Next-generation sequencing (NGS) can resolve enrichment trajectories that colony picking may miss [6,7]. Covalent formats such as SpyDisplay can decouple library production from display assembly and improve modularity [49]. Figure 5 summarizes M13 library construction, target binding and washing, and iterative elution and amplification.

Enrichment is not equivalent to affinity. Transformation bottlenecks and uneven input representation introduce library bias; infectivity, secretion burden, and bacterial growth introduce propagation bias; and target immobilization may denature an antigen or expose non-native surfaces. Fast-growing or multivalent clones can outcompete rare high-affinity binders, whereas sequences that bind plastic, beads, the fragment crystallizable (Fc) region, affinity tags, or blocking agents can appear as false positives. Useful controls include sequencing the naïve and intermediate libraries, parallel negative selections, confirmation with soluble target or target-expressing cells, limited amplification, replicate campaigns, and orthogonal kinetic measurements [6,7,9,10]. Yeast-surface, ribosome, messenger RNA (mRNA), and mammalian cell display offer different trade-offs in accessible diversity, folding and post-translational context, selection modality, and experimental bias (Table 2) [50,51,52,53]. Reported diversity ranges are approximate and depend on delivery, recovery, and quality-control criteria. FACS denotes fluorescence-activated cell sorting.

### 3.3. Drug and Antibody Discovery

Phage display is established for antibody and protein-therapeutic discovery [10]. Its principal advantage is the ability to connect library construction, target-based selection, sequence recovery, and directed optimization. Representative translational outputs are summarized in Figure 4C. The selected sequence, however, remains an early lead until binding, function, and product-quality attributes are confirmed.

Affinity maturation can combine focused mutation with kinetic selection. Kiguchi et al. recovered cortisol-specific scFv variants with association constants (K_a_) of 1.2–2.0 × 10^10^ M^−1^—33- to 56-fold above the parental antibody—corresponding to equilibrium dissociation constants (K_d_) of approximately 50–83 pM [54]. Xu et al. optimized the anti-proprotein convertase subtilisin/kexin type 9 (PCSK9) antibody FAP2M21 to a dissociation rate constant of 4.68 × 10^−6^ s^−1^ [55]. NGS can extend such campaigns from isolated clones to population-level sequence analysis [56].

Phage-display-derived antibodies validate the clinical reach of the technology. Adalimumab was the first fully human antibody discovered by phage display to receive US approval, and additional antibodies have subsequently reached the market across inflammatory disease, oncology, infectious disease, and rare disorders [5]. Selected examples are listed in Table 3. Guided selection can also focus repertoires on a defined epitope [57], whereas membrane-mimetic nanodiscs can preserve difficult targets; this strategy yielded the apelin receptor (APJ)-binding single-domain antibody JN241 (K_d_ = 83 pM), which was further engineered into the agonist JN241-9 [58].

Many selected binders nevertheless fail during development. Common causes include aggregation, self-association, polyspecificity, chemical or conformational instability, poor soluble expression, low manufacturing yield, viscosity at formulation concentration, and immunogenic sequence or structural liabilities. Apparent phage avidity can also disappear when a clone is reformatted as soluble Fab or IgG. Developability assessment should therefore begin during selection and include monovalent affinity, specificity panels, expression yield, thermal and colloidal stability, aggregation and self-interaction, sequence liabilities, formulation stress, and relevant functional assays [59,60].

Computational triage now complements experimental developability testing. Sequence-based pipelines can scan complementarity-determining regions and frameworks for deamidation, isomerization, oxidation, unpaired cysteines, glycosylation motifs, hydrophobic patches, charge asymmetry, and related liabilities. Structure-informed algorithms estimate surface hydrophobicity, aggregation-prone regions, self-interaction, and solubility, whereas major histocompatibility complex class II-binding and T-cell-epitope models provide an initial immunogenicity risk screen. Multi-parameter tools such as the Therapeutic Antibody Profiler compare candidates with clinical-stage antibody distributions and can guide library pruning or re-engineering. Because these predictions remain model- and assay-dependent, they should be confirmed using orthogonal biophysical tests and relevant immunogenicity assays [59,60,61].

In infectious-disease research, phage display has recovered antibodies against simian immunodeficiency virus (SIV) gp120, Ebola virus glycoprotein, and *Staphylococcus aureus* α-hemolysin [62,63,64]. These examples illustrate target breadth but do not substitute for testing neutralization, escape, pharmacology, and safety in disease-relevant models.

The practical endpoint is therefore not the most enriched sequence but the best-supported candidate. Selection data should be integrated with biophysical, structural, functional, manufacturability, and safety evidence before a lead is advanced.

### 3.4. Artificial Intelligence and Data-Driven Design

NGS and deep mutational scanning (DMS) convert display experiments into sequence–function datasets. Enrichment trajectories can identify sequence families, epistatic interactions, and escape-sensitive positions, while DMS measures the effect of many individual or combined mutations [7,65]. These data can train or calibrate models that prioritize a focused library rather than merely rank the final round.

Protein language models (PLMs) can propose mutations that preserve sequence plausibility, and experimental work has shown that general PLMs can guide antibody evolution without target-specific training [66]. In phage-display workflows, machine learning can prioritize complementarity-determining-region (CDR) variants, combine sequence and enrichment features, and flag developability risks before synthesis. A trastuzumab-framework campaign, for example, used AI-designed CDR libraries followed by phage selection to identify human epidermal growth factor receptor 2 (HER2)-binding variants [8].

Structure prediction and generative design provide complementary information. AlphaFold can support structural hypotheses, whereas RFdiffusion can propose new backbones and binding architectures [67,68]. Neither model score is a direct measure of affinity or developability. Prospective designs still require expression, selection or screening, kinetic and functional measurements, and, where needed, structural confirmation. Data leakage, assay-dependent labels, repertoire imbalance, and poorly calibrated confidence estimates remain important sources of error. More broadly, model performance depends on standardized, high-quality experimental datasets: assay noise and sampling bias can distort training labels, and performance may deteriorate when a model is applied to antibody families, scaffolds, targets, or selection conditions outside its training distribution. AI-generated candidates should therefore be treated as experimentally testable hypotheses rather than validated leads.

### 3.5. Vaccines and Immunology

Phage display can map antibody-recognized peptides and identify candidate mimotopes or native epitopes. Screening serum from survivors of tilapia lake virus infection identified Pep3 and the native site S1_399–410_; a vaccine based on the latter produced 81.8% survival in fish [69]. Display of the complete set of open reading frames (ORFeome) from *Streptococcus equi* likewise identified immunogenic surface proteins for vaccine investigation [70].

Phage particles can also deliver antigens. T4 displaying influenza hemagglutinin subunit 1 (HA1) and matrix protein 2 ectodomain (M2e) through small outer capsid (Soc) and highly antigenic outer capsid (Hoc) proteins induced balanced humoral responses and protected mice [71]. Engineered filamentous phages have supported targeted, needle-free severe acute respiratory syndrome coronavirus 2 (SARS-CoV-2) concepts [72], and M13-derived particles have been adapted for lymph-node-targeted neoantigen delivery [73].

Low-cost fermentation, genetic tractability, multivalent presentation, and physical stability are attractive attributes. Immune outcomes nevertheless depend on capsid composition, antigen copy number and orientation, particle dose, innate sensing, tissue distribution, and prior exposure [11,74].

Platform choice is especially important for vaccination. Filamentous phages such as M13 enable non-lytic production and tunable low- or high-valency display but may present conformationally restricted fusions. Lytic T4 or T7 particles can support dense capsid decoration and strong innate stimulation, yet particle assembly, lysis-derived impurities, and payload-specific stability require control. Anti-phage antibodies may accelerate clearance or alter responses after repeated dosing; the direction and magnitude of this effect vary with phage, route, dose, and schedule. Intramuscular, mucosal, inhaled, and targeted lymphatic routes cannot be assumed equivalent. Durability, cellular immunity, antigen-density thresholds, endotoxin removal, biodistribution, and repeat-dose safety therefore require direct evaluation [11,74]. 

Compared with established technologies, phage vaccines offer inexpensive bacterial production, physically robust particles, multivalent antigen display, and potential mucosal delivery without the lipid-nanoparticle formulations commonly used for mRNA vaccines or the mammalian-cell production required for many viral vectors. In contrast, mRNA and viral-vector platforms provide intracellular antigen expression and can elicit strong cellular immunity, whereas protein-subunit vaccines have extensive manufacturing and regulatory precedents and permit precise antigen composition but commonly require adjuvants and repeat dosing. Phage platforms are less clinically mature and face distinctive challenges in antigen-conformation control, endotoxin clearance, anti-phage immunity, biodistribution, dose standardization, and regulatory classification; their advantages are therefore context dependent rather than universal [11,74,75].

## 4. Conclusions

Bacteriophage-derived platforms are most useful when biology and product goals are considered together. λ and N15 remain valuable genetic vehicles; T7 supports lytic expression and capsid-display formats; and M13 offers a mature route from diverse libraries to sequence-defined binders. Across these systems, editing success, selection enrichment, and clinical translation are distinct milestones that require different evidence.

Priority directions include standardized synthetic repertoires, automated microplate panning and screening [76], microfluidic selections that reduce reagent use and improve control over electrophoretic separation [77], and NGS/DMS analysis across selection rounds. Structural methods should be linked to affinity maturation, and AI models should be evaluated in closed loops that propose variants, receive experimental measurements, and update the next library. Developability and formulation filters should enter this workflow before affinity optimization is complete.

Several issues remain unresolved: benchmark datasets are rarely comparable across laboratories; reference standards for library quality and selection reproducibility are lacking; negative results and propagation artifacts are underreported; and predictive models often lack prospective, blinded validation. For therapeutic translation, the field also needs clearer acceptance criteria for polyspecificity, stability, immunogenicity, manufacturing consistency, and—when particles are administered—regulatory control of impurities, biodistribution, and repeat dosing. Addressing these questions will determine whether increasingly sophisticated discovery workflows produce more reliable products rather than merely more candidates.

Clinical translation will also require scalable manufacturing processes compliant with current good manufacturing practice that control particle identity, antigen density, genetic stability, potency, residual host-cell DNA and protein, endotoxin, and batch-to-batch consistency. Regulatory strategies must be matched to whether the phage is used only as a discovery reagent or administered as the product. For administered particles, safety evaluation should address biodistribution, shedding, innate inflammatory responses, anti-phage immunity, off-target effects, and repeat-dose exposure [11,74].

## Figures and Tables

**Table 1:** Decision-oriented comparison of classical phage-derived vector and display systems.

System	Genome/Form	Typical Foreign-DNA Capacity	Replication Mode	Display Strategy	Representative Uses	Principal Advantages	Principal Limitations
λ [4]	Linear dsDNA; circularizes after entry	~10–25 kb in replacement vectors	Lytic or temperate; packaging-dependent	Capsid fusions possible but not the dominant use	Genomic/cDNA libraries; cloning	Defined packaging window; efficient in vitro packaging	Size constraints; lysogeny and host dependence
N15-derived pJAZZ [22]	Hairpin-ended linear dsDNA replicon	Up to ~30 kb demonstrated	TelN/tos-dependent linear-plasmid maintenance	Not primarily a display platform	Unstable, repetitive, or GC-rich DNA	Reduces instability caused by circular plasmids	Specialized host/vector; lower routine throughput
M13/phagemid [9,10]	Circular ssDNA; dsDNA replicative form	Small inserts to antibody-fragment genes; fusion-dependent	Non-lytic infection and extrusion	pIII/pVIII and other coat-protein fusions	Peptide/antibody libraries; affinity selection	Direct genotype–phenotype linkage; mature protocols	Bacterial folding; propagation and valency bias
T7 [9]	Linear dsDNA (~40 kb)	Platform-dependent; peptide to protein inserts	Rapid lytic replication; intracellular capsid assembly	Capsid-protein fusions at format-dependent valency	Protein expression; peptide/protein display	Cytoplasmic expression; robust lytic production	Lysis; platform-specific cargo tolerance; host constraints

**Table 2:** Comparison of display platforms for binder discovery.

Platform	Genotype–Phenotype Linkage	Typical Accessible Diversity (Approx.)	Folding/Modification Context	Selection/Readout	Strengths	Key Limitations
Phage [9,10]	DNA packaged in particle displaying fusion	10^9^–10^11^	Bacterial; limited human post-translational modification	Biopanning; sequencing	Robust, inexpensive, large libraries	Propagation bias; avidity; target-format artifacts
Yeast surface [50]	Plasmid/host genotype linked to cell-surface fusion	Transformation-limited; typically below phage	Eukaryotic folding; yeast glycosylation	FACS with quantitative gates	Expression and binding measured per cell	Smaller libraries; non-human glycosylation
Ribosome [51]	Noncovalent mRNA–ribosome–protein complex	≥10^12^	Cell-free; tunable translation mixture	In vitro affinity selection	Very large libraries; no transformation	Fragile complexes; limited cellular quality control
mRNA [52]	Covalent mRNA–puromycin–protein fusion	10^12^–10^13^	Cell-free; tunable conditions	In vitro affinity selection	Large libraries; covalent linkage	RNA handling; amplification and translation bias
Mammalian cell [53]	Integrated or episomal genotype linked to surface protein	~10^6^ in established workflows	Near-native mammalian folding and modification	FACS; functional cell assays	Direct expression; product-quality screens	Cost, slower workflows, smaller libraries

**Table 3:** Selected clinically approved antibodies derived from or engineered using phage display.

Antibody	Target	Initial Approved Indication	Initial US Approval	Displayed Format	Library Type/Strategy	Final Format
Adalimumab [5]	TNF-α	Rheumatoid arthritis	2002	scFv	Guided selection using a naïve human library (CAT)	IgG1-κ
Ranibizumab [5]	VEGF-A	Neovascular age-related macular degeneration	2006	Fab	Focused affinity-maturation libraries	Fab
Belimumab [5]	BAFF/BLyS	Systemic lupus erythematosus	2011	scFv	Naïve human library (CAT)	IgG1-λ
Raxibacumab [5]	Anthrax protective antigen	Inhalational anthrax	2012	scFv	Naïve human library (CAT)	IgG1-λ
Ramucirumab [5]	VEGFR2	Advanced gastric cancer	2014	Fab	Naïve human library (Dyax)	IgG1
Necitumumab [5]	EGFR	Metastatic squamous non-small-cell lung cancer	2015	Fab	Naïve human library (Dyax)	IgG1-κ
Avelumab [5]	PD-L1	Metastatic Merkel cell carcinoma	2017	Fab	Naïve human library (Dyax)	IgG1-λ
Guselkumab [5]	IL-23 p19	Moderate-to-severe plaque psoriasis	2017	Fab	Synthetic human library (HuCAL GOLD)	IgG1-λ
Lanadelumab [5]	Plasma kallikrein	Hereditary angioedema	2018	Fab	Naïve human library (Dyax)	IgG1-κ
Emapalumab [5]	IFN-γ	Primary hemophagocytic lymphohistiocytosis	2018	scFv	Naïve human library (CAT)	IgG1-λ

Approval years, phage-display formats, library types, and final therapeutic formats are summarized from Alfaleh et al. [5]. BAFF/BLyS, B-cell activating factor/B-lymphocyte stimulator; EGFR, epidermal growth factor receptor; IFN-γ, interferon gamma; PD-L1, programmed death-ligand 1; TNF-α, tumor necrosis factor alpha; VEGF-A, vascular endothelial growth factor A; VEGFR2, vascular endothelial growth factor receptor 2. CAT, Cambridge Antibody Technology; Fab, antigen-binding fragment; HuCAL, Human Combinatorial Antibody Library; IgG, immunoglobulin G; scFv, single-chain variable fragment.

## Data Availability

No new data were created or analyzed in this study. Data sharing is not applicable to this article.

## Abbreviations

The following abbreviations are used in this manuscript:

AI	artificial intelligence
APJ	apelin receptor
BAFF/BLyS	B-cell activating factor/B-lymphocyte stimulator
CAT	Cambridge Antibody Technology
cDNA	complementary DNA
CDR	complementarity-determining region
cGAMP	cyclic GMP–AMP
CRISPR–Cas	clustered regularly interspaced short palindromic repeats–CRISPR-associated system
DMS	deep mutational scanning
DNA	deoxyribonucleic acid
dsDNA	double-stranded DNA
dsRNA	double-stranded RNA
*E. coli*	*Escherichia coli*
EGFR	epidermal growth factor receptor
Fab	antigen-binding fragment
FACS	fluorescence-activated cell sorting
Fc	fragment crystallizable
HA1	hemagglutinin subunit 1
HER2	human epidermal growth factor receptor 2
HuCAL	Human Combinatorial Antibody Library
Hoc	highly antigenic outer capsid protein
IgG	immunoglobulin G
IFN-γ	interferon gamma
IL-23	interleukin 23
K_a_	association constant
K_d_	equilibrium dissociation constant
LPS	lipopolysaccharide
M2e	matrix protein 2 ectodomain
mRNA	messenger RNA
NGS	next-generation sequencing
ORF	open reading frame
ORFeome	complete set of open reading frames
PARIS	phage anti-restriction-induced system
PCR	polymerase chain reaction
PCSK9	proprotein convertase subtilisin/kexin type 9
PD-L1	programmed death-ligand 1
PHEIGES	PHage Engineering by In vitro Gene Expression and Selection
PLM	protein language model
pIII	minor coat protein III
pVIII	major coat protein VIII
RNA	ribonucleic acid
scFv	single-chain variable fragment
SARS-CoV-2	severe acute respiratory syndrome coronavirus 2
SIV	simian immunodeficiency virus
SMART	splitting, modifying, assembling, and rebooting
Soc	small outer capsid protein
ssDNA	single-stranded DNA
ssRNA	single-stranded RNA
TNF-α	tumor necrosis factor alpha
tRNA	transfer RNA
TXTL	cell-free transcription–translation
VEGF-A	vascular endothelial growth factor A
VEGFR2	vascular endothelial growth factor receptor 2
